# Impact of the Family‐Centered Empowerment Model on Health‐Promoting Lifestyle and Psychological Well‐Being in Patients With Multiple Sclerosis: A Randomized Controlled Clinical Trial

**DOI:** 10.1002/hsr2.73423

**Published:** 2026-10-26

**Authors:** Leila Ranjber, Mostafa Bijani, Azizallah Dehghan, Roya Dokoohaki, Azam Zare

**Affiliations:** ^1^ Student Research Committee, Fasa University of Medical Sciences Fasa Iran; ^2^ Department of Medical Surgical Nursing School of Nursing, Fasa University of Medical Sciences Fasa Iran; ^3^ NonCommunicable Diseases Research Center (NCDRC), Fasa University of Medical Sciences Fasa Iran; ^4^ Community Based Psychiatric Care Research Center Nursing and Midwifery School of Nursing, Shiraz University of Medical Sciences Shiraz Iran

**Keywords:** family‐centered empowerment model, health‐promoting lifestyle, multiple sclerosis, psychological well‐being

## Abstract

**Background and Aim:**

Multiple sclerosis (MS) is a chronic neurological disease that can affect patients’ health‐promoting lifestyle and psychological well‐being and may also place substantial demands on family caregivers. This study aimed to evaluate the effect of a family‐centered empowerment intervention based on the Family‐Centered Empowerment Model (FCEM) on health‐promoting lifestyle and psychological well‐being among patients with MS, with the active involvement of their primary family caregivers.

**Methods:**

This randomized controlled clinical trial was conducted from February to April 2025 at an MS clinic in Fars Province, southern Iran. It included 70 patient‐caregiver dyads, comprising 70 patients with MS and their primary family caregivers. The dyads were randomly assigned to the intervention or control group, with 35 dyads in each group. Patients and caregivers in the intervention group took part in a structured family‐centered empowerment program based on the four‐stage FCEM. The program consisted of five to seven face‐to‐face educational sessions attended jointly by the patients and their primary family caregivers. The control group continued to receive routine care. Health‐promoting lifestyle and psychological well‐being were assessed at baseline, immediately after the intervention, and at the 2‐month follow‐up.

**Results:**

At baseline, the two groups did not differ significantly in health‐promoting lifestyle or psychological well‐being (*p* > 0.05). Following the intervention, the mean health‐promoting lifestyle score was 126.40 ± 25.18 in the intervention group and 102.62 ± 25.18 in the control group (*p* < 0.001). At the 2‐month follow‐up, the corresponding scores were 124.34 ± 22.39 and 104.22 ± 20.23, respectively (*p* < 0.001). A similar pattern was observed for psychological well‐being. Immediately after the intervention, the mean score was 73.68 ± 13.83 in the intervention group, compared with 59.62 ± 10.24 in the control group (*p* < 0.001). At 2 months, the scores were 71.51 ± 12.82 and 61.34 ± 11.55, respectively (*p* < 0.001). Within the intervention group, scores increased significantly from baseline to both the post‐intervention assessment and the 2‐month follow‐up (*p* < 0.001). Changes in the control group were comparatively small.

**Conclusion:**

A family‐centered empowerment intervention based on the FCEM was associated with greater improvements in health‐promoting lifestyle and psychological well‐being among patients with MS compared with routine care. Involving primary family caregivers in MS education and self‐management support may be a useful component of family‐centered care.

**Iranian Registry of Clinical Trials:** IRCT registration number: IRCT20190917044802N12. **Registration date:** 10/01/2025.

## 1 Introduction

Multiple sclerosis (MS) is a chronic autoimmune disorder characterized by immune‐mediated damage to the central nervous system [1]. It most commonly presents between the ages of 20 and 40 years, with a mean age of onset of approximately 30 years. Epidemiological evidence indicates that its prevalence among women is approximately two to three times higher than that observed in men [2]. Globally, nearly 2.8 million individuals are affected by this condition. Within the Middle East, Iran reports the highest prevalence rate; according to recent data from the Iranian MS Association, approximately 100 in 100000 which is high [3, 4, 5].

The clinical course of MS is often marked by progressive disability, encompassing a wide range of sensory and motor impairments [2]. As the disease advances, many patients experience a decline in their capacity to perform activities of daily living independently, resulting in increasing reliance on caregivers, most commonly immediate family members [6]. Notably, the caregiving demands associated with MS differ considerably from those observed in other chronic illnesses. Owing to the inherently unpredictable and heterogeneous trajectory of the disease, caregivers frequently face uncertainty in anticipating exacerbations, relapses, and even day‐to‐day fluctuations in functional status [7]. Addressing the educational needs of family caregivers, who constitute a central component of the support system for individuals with MS, is essential for the effective implementation of empowerment‐based interventions [8]. Since its formal introduction into chronic disease management in 2003, the concept of empowerment has evolved into a dynamic, multidimensional construct encompassing cognitive, social, and functional domains, ultimately enabling patients to exercise greater control over their condition [9].

The Family‐Centered Empowerment Model (FCEM), developed by Fatemeh Alhani in 2003 for use among patients with chronic diseases in Iran, is grounded in strengthening the family as a cohesive and functional unit. By actively engaging both patients and their caregivers, the model seeks to enhance self‐efficacy, improve quality of life, and alleviate caregiving burden [10]. In practice, FCEM is operationalized through several interrelated mechanisms, including increasing perceived threat through heightened awareness of severity and susceptibility, reinforcing self‐efficacy through skill acquisition, promoting self‐esteem via self‐help and self‐regulation strategies, and incorporating continuous evaluation processes [11]. Earlier studies have reported favorable effects of FCEM and other empowerment‐oriented interventions on health‐related and psychosocial outcomes in people with chronic diseases [12]. Studies involving people with MS have also reported findings related to empowerment, family involvement, and disease management. Homayouni et al. [13], for example, found that a healthy lifestyle empowerment program for family caregivers of patients with MS improved adherence to health‐promoting behaviors and reduced caregiver burden [13]. Mousaei et al. [14] similarly reported lower caregiving burden among family caregivers of individuals with MS who received support based on the FCEM. Bayomi and Metwaly [15] examined a health‐promotion program for patients with MS and reported improved adherence to the therapeutic regimen. Bayat et al. [16] found that a patient‐centered empowerment intervention delivered through telenursing had the potential to improve self‐management among people with MS. Raji et al. [17] also reported beneficial effects of a therapeutic patient education program on quality of life in individuals with MS. These studies suggest that educational and empowerment‐based interventions may benefit self‐management, health‐promoting behaviors, caregiver‐related outcomes, and quality of life in people affected by MS.

Health‐promoting lifestyle is particularly relevant to individuals living with MS because daily health behaviors may influence physical functioning, disease management, and psychological adaptation to chronic illness [18, 19]. Previous research has indicated an association between engagement in health‐promoting behaviors and mental health outcomes among individuals with MS [20]. Psychological well‐being (PWB) is also an important dimension of health in this population because living with a chronic and potentially disabling disease may affect emotional adjustment, social relationships, sense of purpose, and perceived control over daily life [21, 22]. Consequently, interventions that simultaneously address health‐promoting behaviors and PWB may provide a more comprehensive approach to supporting patients with MS.

## 2 Research Gap and Significance of the Study

Despite the growing body of work on empowerment and educational interventions in MS, some gaps remain. Previous studies have often examined individual outcomes, such as self‐management, caregiver burden, adherence to health‐promoting behaviors, or quality of life, without considering health‐promoting lifestyle and PWB together. Family participation is central to FCEM, yet relatively few studies have included both patients with MS and their primary family caregivers in the intervention while assessing behavioral and psychological outcomes at the same time. The effect of a family‐centered empowerment intervention on both health‐promoting lifestyle and PWB in patients with MS therefore remains unclear. The present randomized controlled clinical trial was conducted to determine whether a family‐centered empowerment intervention based on FCEM could improve health‐promoting lifestyle and PWB among patients with MS when their primary family caregivers were actively involved. The findings may provide a basis for developing more integrated family‐centered educational approaches to MS management.

## 3 Methods

This study was designed as a randomized controlled clinical trial with a non‐blinded design, including intervention and control groups. The study was conducted between February and April 2025 at an MS clinic in Fars Province, southern Iran. The study aimed to evaluate the effectiveness of a family‐centered empowerment intervention based on the FCEM in improving health‐promoting lifestyle and PWB among patients with MS, with the active involvement of their primary family caregivers. **CONSORT** (Consolidated Standards of Reporting Trials) checklist was used to determine the quality of randomized controlled trials (Supporting Information S1: CONSORT checklist).

### 3.1 Participants and Eligibility Criteria

The study population consisted of patient–caregiver dyads. Each dyad included one patient with a confirmed diagnosis of MS and one primary family caregiver who was involved in the patient's daily care and support.

### 3.2 Inclusion Criteria

Patients were eligible if they had a confirmed diagnosis of MS established by a neurologist, had a minimum disease duration of 6 months, possessed sufficient literacy to complete the study questionnaires, expressed willingness to participate, and provided informed consent. Patients were also required to have had no previous participation in a similar educational intervention during the 6 months preceding the study. Primary family caregivers were eligible if they were identified by the patient as the main family member providing care and support, were willing to participate in the educational intervention, possessed sufficient literacy to participate in the study procedures, and provided informed consent.

### 3.3 Exclusion Criteria

Patients were excluded if they experienced a clinical exacerbation during the study period, did not attend the educational sessions, with nonattendance defined as missing more than two sessions, or voluntarily withdrew from the study. Patients with documented psychological or cognitive impairments were also excluded, as were those with concurrent chronic conditions that might have confounded the study outcomes. Caregivers were excluded if they missed more than two training sessions or voluntarily discontinued their participation at any stage of the study.

### 3.4 Sample Size Estimation

The required sample size was calculated based on a statistical power of 90% and a significance level of 0.05. Based on the findings of Rahimi Kordshooli et al. [23], a minimum of 31 patients per group was estimated to be required. To compensate for potential attrition and maintain adequate statistical power, the sample size was increased to 35, patient–caregiver dyads per group. The final study population consisted of 70 patients with MS and their 70 primary family caregivers, forming 70 patient‐caregiver dyads. Thirty‐five dyads were assigned to the intervention group and 35 to the control group.

$$n=\frac{{\left(Z_{1-\frac{\alpha }{2}+Z_{1-\beta }}\right)}^{2}\left(s_{1}^{2}+s_{2}^{2}\right)}{d^{2}}$$

### 3.5 Recruitment Procedure

Potentially eligible patients with MS were recruited in person at the study site. Initially, 100 patients were approached through convenience sampling. The researcher explained the study, including its objectives and procedures, and assessed each patient's eligibility according to the predetermined inclusion and exclusion criteria. Patients who agreed to participate were asked to identify their primary family caregiver. The nominated caregiver was then contacted in person and informed about the study, the expected participation requirements, and the educational intervention. The patient and caregiver were subsequently considered one patient‐caregiver dyad for recruitment and intervention purposes. After the eligibility criteria were applied, 30 patients were excluded, leaving 70 eligible patient‐caregiver dyads. Enrollment proceeded after written informed consent had been obtained from both members of each dyad, namely, the patient and the corresponding primary family caregiver.

### 3.6 Random Allocation

The 70 eligible patient–caregiver dyads were randomly allocated to either the intervention or control group. Seventy allocation cards were prepared, including 35 cards labeled “A” and 35 cards labeled “B.” Each card was placed in an opaque, sealed envelope to ensure allocation concealment. Each enrolled patient–caregiver dyad selected one sealed envelope at random. Dyads assigned to cards labeled “A” were allocated to the control group, whereas those assigned to cards labeled “B” were allocated to the intervention group. Accordingly, each study group included 35, patient–caregiver dyads. A CONSORT flow diagram illustrating the recruitment, allocation, follow‐up, and analysis of the patient–caregiver dyads is presented in Figure 1.

**Figure 1:**
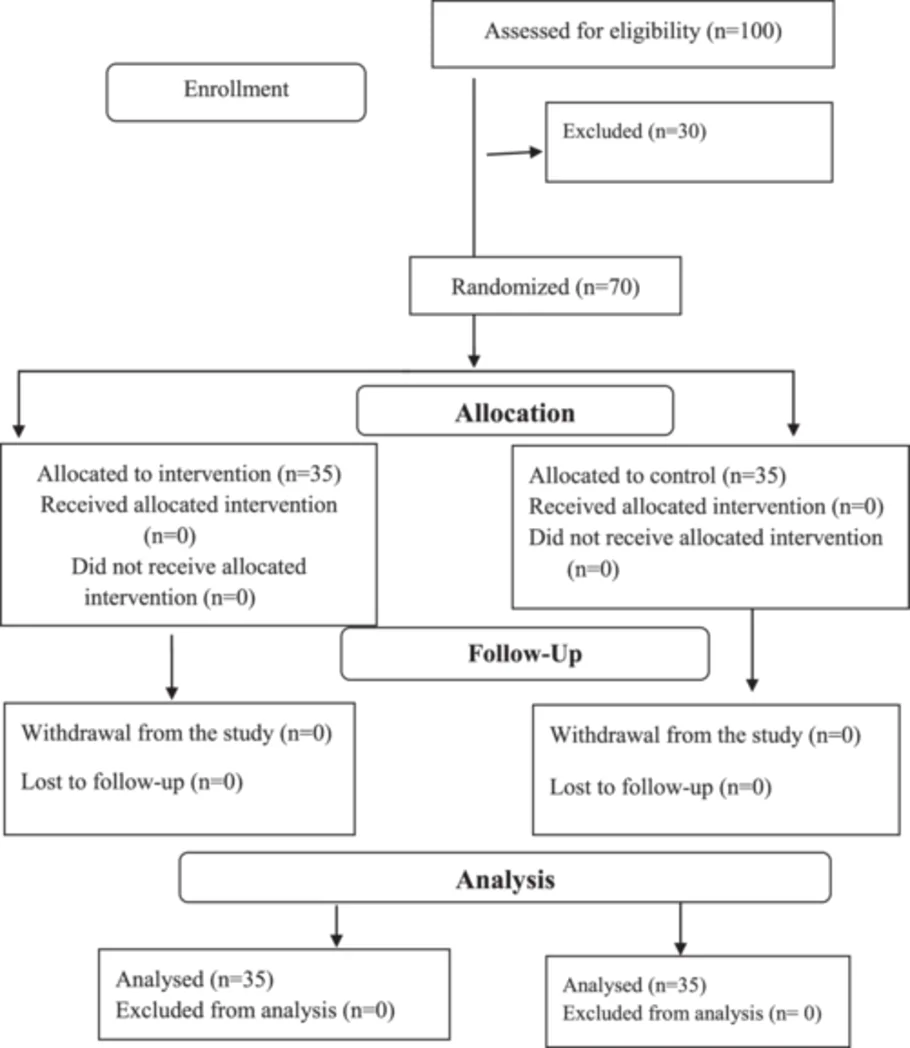
Figure 1 displays consort flow diagram throughout the study.

### 3.7 Data Collection Tools

Data were collected using a structured set of instruments, including a Demographic Information Questionnaire, Walker's Health‐Promoting Lifestyle Profile (HPLP), and Ryff's PWB Scale.

### 3.8 Demographic Information Questionnaire

The demographic questionnaire was used to collect sociodemographic and clinical characteristics. For patients with MS, the questionnaire included age, gender, marital status, occupational status, educational level, place of residence, and duration of illness. The caregiver questionnaire included age, gender, marital status, occupation, educational level, place of residence, and the caregiver's relationship to the patient.

### 3.9 Health‐Promoting Lifestyle Profile (Walker, Sechrist, and Pender, 1987)

The HPLP, developed by Walker, Sechrist, and Pender (1987), is a multidimensional instrument designed to assess health‐promoting behaviors. It comprises 52 items distributed across six domains: self‐actualization, nutrition, physical activity, health responsibility, stress management, and interpersonal support. Responses are recorded on a 4‐point Likert scale ranging from “Always” (score = 4) to “Never” (score = 1), yielding a total score between 52 and 208, with higher scores indicating more favorable health‐promoting lifestyle patterns. The psychometric properties of the HPLP have been evaluated within the Iranian context by Mohammadi Zeidi et al. [24], who reported Cronbach's alpha coefficients for the subscales ranging from 0.64 to 0.91. Furthermore, Sepah Mansour and Bagheri [25] confirmed the overall reliability of the instrument, reporting a Cronbach's alpha coefficient of 0.91.

### 3.10 Psychological Well‐Being Scale

The PWB Scale, developed by [26] Carol D. Ryff, was originally constructed as a 42‐item instrument. A shorter version comprising 18 items (Ryff and Keyes 1995) was subsequently developed, encompassing six core dimensions: autonomy, environmental mastery, personal growth, positive relations with others, purpose in life, and self‐acceptance. The instrument employs a 7‐point Likert scale ranging from 1 (*strongly agree*) to 7 (*strongly disagree*), with higher scores reflecting greater PWB. Extensive psychometric evaluations conducted by Ryff have demonstrated high internal consistency across diverse populations, with Cronbach's alpha coefficients typically ranging from 0.83 to 0.93. Specifically, reported reliability coefficients include self‐acceptance (0.93), positive relations with others (0.91), autonomy (0.86), environmental mastery (0.90), purpose in life (0.90), and personal growth (0.87). Moreover, Nazarpoour et al. [27] reported a Cronbach's alpha coefficient of 0.79 for this instrument, further supporting its reliability.

### 3.11 Educational Intervention

#### 3.11.1 Interventional Group

The intervention administered to the intervention group was structured according to the four‐stage framework of the FCEM and was tailored to the educational needs of each patient–caregiver dyad. Before implementation of the intervention, a comprehensive needs assessment was conducted to identify the specific educational needs of patients and their primary family caregivers. The assessment addressed topics including the nature and classification of MS, possible causes and clinical manifestations, diagnostic procedures, available treatment modalities, medication management and potential adverse effects, nutrition, physical activity, and psychological concerns such as depression. The educational content was developed based on the identified needs. Content validity was evaluated by an expert panel consisting of 10 PhD‐qualified nursing education specialists, two neurologists, and the educational supervisor affiliated with the study setting. The first stage of the intervention, which focused on enhancing perceived threat, was delivered in five groups. Subsequent stages were delivered either individually to one patient–caregiver dyad or in small groups consisting of two to three patient–caregiver dyads. All 35 intervention dyads received the complete educational program based on the FCEM. Based on the initial needs assessment, each group participated in five to seven face‐to‐face educational sessions. Each session lasted approximately 45–60 min, and sessions were scheduled at intervals of 5–7 days according to participant availability. All sessions were conducted in the hospital conference room in coordination with hospital administrators and the MS Association. The implementation of the FCEM stages followed the methodological frameworks described by Shirvani et al. [28] and Masoodi et al. [29] (Supporting Information S2: Education sessions).

#### 3.11.2 Control Group

The control group did not receive any structured or supplementary training; rather, they were provided solely with an educational booklet. Outcome assessments were conducted at three predefined time points: prior to the initiation of the intervention, immediately following its completion, at the 2‐month follow‐up, and at the conclusion of the study period. At each of these intervals, patient–caregiver in both the intervention and control groups completed the study questionnaires, with guidance and support provided by the first author (LR).

### 3.12 Informed Consent

Before the intervention began, the researcher met with the patients and caregivers to explain the study objectives and procedures, anticipated benefits and potential risks, the confidentiality of the information collected, and their right to withdraw at any time without adverse consequences. Written informed consent was then obtained from all patients and their primary family caregivers in both study groups at the MS clinic. Consent from both members of each dyad was obtained before formal enrollment and before the intervention began.

### 3.13 Outcome Assessment

The primary outcomes were health‐promoting lifestyle and PWB among patients with MS. Measurements were collected at three pre‐specified time points: baseline, immediately after completion of the intervention, and at the 2‐month follow‐up. At each time point, participants in both groups completed the HPLP and PWB questionnaires. The questionnaires were administered under standardized conditions. When participants had questions about how to complete a questionnaire, the first author (LR) provided clarification. The same assessment procedures were used at all three time points.

### 3.14 Data Analysis

All statistical analyses were performed using SPSS software (version 25). Continuous variables were summarized using descriptive statistics, including means and standard deviations, whereas categorical variables were reported as frequencies and percentages. The normality of quantitative data was assessed using the Shapiro–Wilk test. Baseline characteristics between the two groups were compared using either the independent‐samples *t*‐test or the Mann–Whitney *U*‐test, as appropriate based on data distribution. Additionally, repeated‐measures analysis of variance (RM‐ANOVA) was employed to evaluate changes over time in the mean scores of health‐promoting lifestyle and PWB, both within and between groups. A *p*‐value of less than 0.05 was considered statistically significant.

### 3.15 Ethical Considerations

The present study was conducted in strict accordance with the principles of the revised Declaration of Helsinki, an internationally recognized framework providing ethical guidance for research involving human participants. Written informed consent was obtained from all participants prior to enrollment, ensuring their full understanding of the study objectives and their unequivocal right to withdraw at any stage. The confidentiality of participants’ personal information was rigorously maintained, and all procedures were clearly communicated to ensure both anonymity and data protection. Ethical approval was granted by the Research Ethics Committees of Fasa University of Medical Sciences, Fars, Iran (code: IR.FUMS.REC.1403.115).

## 4 Results

As presented in Table 1, the mean age (± standard deviation) of patients in the intervention and control groups was 39.54 ± 8.76 and 42.77 ± 9.76 years, respectively. Of the 70 patients included in the study, 52 (74.3%) were women and 18 (25.7%) were men. Statistical analyses revealed no significant differences between the intervention and control groups with respect to demographic and baseline characteristics, indicating that the two groups were comparable at baseline. As shown in Table 2, the mean HPLP score did not differ significantly between the intervention and control groups at baseline (94.48 ± 9.98 vs. 98.00 ± 13.42, respectively; *p* = 0.218). However, significant between‐group differences were observed immediately after the intervention and at the 2‐month follow‐up. Immediately after the intervention, the mean HPLP score increased to 126.40 ± 25.18 in the intervention group compared with 102.62 ± 25.18 in the control group (*p* < 0.001; effect size = 1.12). At the 2‐month follow‐up, the corresponding mean scores were 124.34 ± 22.39 and 104.22 ± 20.23, respectively (*p* < 0.001; effect size = 0.942). Within‐group analyses showed significant changes over time in both groups. In the intervention group, the HPLP score increased significantly from baseline to immediately after the intervention (*p* < 0.001) and remained significantly higher than baseline at the 2‐month follow‐up (*p* < 0.001). In the control group, significant changes were also observed over time, although the magnitude of change was smaller (*p* = 0.012) immediately after the intervention and (*p* = 0.006) at the 2‐month follow‐up). The mean HPLP score in the intervention group increased by 18.20 points from baseline to immediately after the intervention and by 16.02 points from baseline to the 2‐month follow‐up, compared with increases of 4.37 and 6.08 points, respectively, in the control group; the between‐group differences in change were statistically significant (*p* < 0.001 for both comparisons).

**Table 1:** Distribution of demographic variables in MS patients in terms of control and intervention group.

Variable	Intervention group	Control group	*p*‐value
Age (mean ± SD)	39.54 ± 8.76	42.77 ± 9.76	0.150*
Disease history (mean ± SD)	9.94 ± 6.38	10.62 ± 6.74	0.434*
Gender (number & percent)			
Man	6 (17.1%)	12 (34.3%)	0.101**
Woman	29 (82.9%)	23 (65.7%)	
Marital status (number & percent)			
Single/divorced/widow (widower)	7 (20%)	9 (25.7%)	0.565**
Married	28 (80%)	26 (74.3%)	
Education (number & percent)			
Under diploma	12 (34.28%)	13 (37.14%)	0.376**
Diploma and higher	23 (65.71%)	22 (62.85%)	
Main caregiver (number & percent)			
Spouse	16 (45.7%)	16 (45.7%)	0.773**
Mother	10 (28.6%)	7 (20%)	
Child	7 (20%)	8 (22.9%)	
Other ones (sister/brother/father)	2 (5.7%)	4 (11.4%)	
Caregiver's gender			
Man	11 (31.4%)	10 (28.6%)	0.794**
Woman	24 (68.6%)	25 (71.4%)	
Caregiver's age (mean ± SD)	41.20 ± 11.68	42.05 ± 13.56	0.320*
Caregiver's education (number & percent)			
Under diploma	7 (22.9%)	9 (25.7%)	0.857**
Diploma & higher	28 (77.1%)	26 (74.3%)	

* Independent sample *t*‐test.

** Chi square test.

**Table 2:** Mean and standard deviation of health‐promoting lifestyle in control and intervention groups.

	Intervention (*N* = 35)		Control (*N* = 35)		*p*‐value^a^	Effect size
	Mean	SD	Mean	SD	—	
Before the intervention	94.48	9.98	98	13.42	0.218	−0.297
Immediately after the intervention	126.40	25.18	102.62	25.18	< 0.001	1.12
*p*‐value^b^	< 0.001		0.012			
After 2 months	124.34	22.39	104.22	20.23	< 0.001	0.942
*p*‐value^b^	< 0.001		0.006		—	—

**Difference**	**Mean**	**SD**	**Mean**	**SD**	***p*‐value** c	
Immediately after/before the intervention	18.2	11.09	4.37	15.29	< 0.001	13.36
After 2 months/Immediately after the intervention	−2.17	5.55	1.71	5.21	0.004	12.44
After 2 months/Before the intervention	16.02	10.72	6.08	13.94	< 0.001	12.44

^a^ Independent sample *t*‐test for pre‐intervention. ANOVA for immediately after intervention and 2 months later.

^b^ Repeated measures ANOVA.

^c^ Mann–Whitney test for differences.

As shown in Table 3, no statistically significant difference was observed between the intervention and control groups in mean PWB scores at baseline. Immediately after the intervention, the mean PWB score was 73.68 ± 13.83 in the intervention group and 59.62 ± 10.24 in the control group (*p* < 0.001; effect size = 1.15). At the 2‐month follow‐up, the corresponding mean scores were 71.51 ± 12.82 and 61.34 ± 11.55, respectively (*p* < 0.001; effect size = 0.833). Within‐group analyses demonstrated significant changes over time in both groups. In the intervention group, PWB increased significantly from baseline to immediately after the intervention and remained significantly higher than baseline at the 2‐month follow‐up (*p* < 0.001 for both comparisons). In the control group, significant changes over time were also observed (*p* = 0.030 immediately after the intervention and (*p* = 0.013) at the 2‐month follow‐up). The mean PWB score in the intervention group increased by 31.91 points from baseline to immediately after the intervention and by 29.85 points from baseline to the 2‐month follow‐up, compared with increases of 4.62 and 6.22 points, respectively, in the control group. The between‐group difference in change from baseline to immediately after the intervention was statistically significant (*p* < 0.001), whereas the difference in change between the 2‐month and immediate post‐intervention assessments was not statistically significant (*p* = 0.119).

**Table 3:** Mean and standard deviation of psychological well‐being in control and intervention groups.

	Intervention (*N* = 35)		Control (*N* = 35)		*p*‐value^a^	Effect size
	Mean	SD	Mean	SD		
Before	55.25	9.15	55.28	16.95	0.944	0.017
Immediately after the intervention	73.68	13.83	59.62	10.24	< 0.001	1.15
*p*‐value^b^	< 0.001		0.03			
After 2 months	71.51	12.82	61.34	11.55	< 0.001	0.833
*p*‐value^b^	< 0.001		0.013			

**Difference**	**Mean**	**SD**	**Mean**	**SD**	***p*‐value** c	
Immediately after/before the intervention	31.91	20.88	4.62	10.27	< 0.001	1.65
After 2 months/Immediately after the intervention	−2.05	7.91	1.6	12.58	0.119	−0.471
After 2 months/Before the intervention	29.85	17.61	6.22	7.59	< 0.001	1.54

^a^ Independent sample *t*‐test for before intervention. ANOVA for immediately after intervention and after 2 months, intervention.

^b^ Repeated measures ANOVA.

^c^ Mann–Whitney test for differences.

## 5 Discussion

This randomized controlled clinical trial examined the effects of a family‐centered empowerment intervention based on the FCEM on health‐promoting lifestyle and PWB among people with MS. Primary family caregivers participated alongside the patients. Compared with the control group, participants in the intervention group had higher HPLP and PWB scores immediately after the intervention and at the 2‐month follow‐up. The difference was particularly evident for PWB, with between‐group effect sizes of 1.15 immediately after treatment and 0.833 at 2 months. Although the scores decreased slightly between the post‐intervention assessment and the 2‐month follow‐up, they remained considerably higher than the baseline scores in the intervention group. These findings indicate that the family‐centered empowerment intervention was associated with improvements in health‐promoting lifestyle and PWB that remained evident 2 months after the intervention. The improvement in health‐promoting lifestyle is consistent with findings from previous studies of educational and empowerment‐based interventions in people with MS. Rooddehghan et al. [30], for example, reported that an 8‐week self‐care education program involving patients and, in one intervention arm, a family member significantly improved quality of life compared with the control condition. After the intervention, quality‐of‐life scores were 53.16 ± 15.19 in the patient‐centered group and 56.03 ± 14.40 in the family‐centered group, compared with 52.48 ± 21.20 in the control group (*p* < 0.001) [30]. Homayouni et al. [13] likewise found, in a study of family caregivers of patients with MS, that adherence to health‐promoting behaviors in the intervention group increased from 123.53 ± 14.01 at baseline to 148.06 ± 15.04 at the second follow‐up, while caregiver burden decreased significantly. These studies differed from the present study in their populations and intervention formats, but their findings are broadly consistent with the possibility that education combined with family involvement can support healthier behaviors in people affected by MS. Bayat et al. [16] more recently reported that a patient‐centered empowerment intervention delivered through telenursing significantly improved self‐management in people with MS. Post‐intervention self‐management scores were 91.62 ± 11.14 in the intervention group and 88.51 ± 11.27 in the control group, with a medium effect size (Cohen's *d* = 0.56) [16]. The present study adds to this literature by examining health‐promoting lifestyle and PWB together within a family‐centered intervention. The findings should also be considered in light of the mixed evidence on family‐centered empowerment among caregivers of patients with MS. Mousaei et al. [14] evaluated an FCEM‐based support intervention involving 60 family caregivers of patients with MS. Caregivers in the intervention group reported lower caregiver‐burden scores after treatment, but the time‐by‐treatment interaction was not statistically significant (*p* = 0.053), so the intervention effect was not conclusive [14]. The difference between those findings and the present results may be related to differences in the outcomes assessed, intervention intensity, follow‐up duration, or characteristics of the study populations. In the present study, the patient‐caregiver dyad was the unit of intervention, and the program focused on health‐promoting lifestyle and PWB rather than caregiver burden alone. These differences in study design and outcomes should be considered when comparing the findings. One possible explanation for the improvement in health‐promoting lifestyle is the way FCEM approaches education. Education in this model is not limited to the transfer of information; participants’ needs and active involvement are also part of the process. In this study, participants received education about MS and its management, medication use and adverse effects, nutrition, physical activity, and psychological concerns. Their needs were assessed, and family caregivers took part in learning and collaborative problem solving. This may have helped patients develop greater disease‐related knowledge, self‐efficacy, perceived control, and motivation to adopt healthier behaviors. Family caregiver involvement may also have made it easier to continue these behaviors at home through practical help, support with treatment recommendations, and emotional support. The improvement in HPLP may therefore reflect both what patients learned during the sessions and how that knowledge was incorporated into their daily lives.

The improvement in PWB is also relevant to people living with MS. Concerns about disease progression, functional limitations, persistent symptoms, changes in family and social roles, and the demands of long‐term disease management may all affect PWB. A family‐centered empowerment approach addresses several of these issues within the same intervention. Greater knowledge of the disease, a clearer understanding of treatment and self‐care, increased self‐efficacy, and the involvement of a trusted family caregiver may help patients feel more‐able to manage the challenges associated with MS. Family members may also provide reassurance and practical assistance, which may support autonomy, environmental mastery, and adaptive coping. Studies using FCEM in other chronic disease populations have similarly reported improvements in psychological and mental health‐related outcomes, including among patients recovering from myocardial infarction and older women. These populations differ from people with MS, however, so those findings are better considered contextual evidence than directly comparable results. The results of the present study also raise the possibility that family‐centered empowerment may affect psychological adaptation in addition to health‐related behaviors. In the intervention group, the mean PWB score increased from 55.25 at baseline to 73.68 immediately after the intervention and was 71.51 at the 2‐month follow‐up. The score therefore remained relatively high after 2 months, although it was somewhat lower than immediately after the intervention. This pattern may indicate that some of the improvement persisted after the educational sessions ended. At the same time, the decline between the two assessments suggests that additional support may be needed if these gains are to be maintained over longer periods. Future studies could examine whether periodic educational follow‐up, telephone support, or digitally delivered reinforcement helps maintain the effects of family‐centered empowerment.

### 5.1 Strengths and Limitations

The study has several methodological strengths. The randomized controlled design reduced the likelihood of systematic baseline differences between the intervention and control groups. Patients and caregivers were also treated as patient‐caregiver dyads, which was consistent with the family‐centered nature of the intervention. Assessments were conducted at baseline, immediately after treatment, and during follow‐up, allowing changes over time to be examined. The intervention was structured around the FCEM framework and adapted to the educational needs identified among the participating patients and caregivers.

Several limitations should nevertheless be considered. The sample consisted of only 70 patient‐caregiver dyads, which limits the precision of the estimates and the generalizability of the findings. In addition, all participants were recruited from a single MS clinic. Patients and caregivers receiving care in other geographical areas or healthcare systems may have different experiences, and the present findings may not fully represent those populations. Blinding was also not feasible for participants or the intervention provider because of the nature of the educational intervention. Performance bias may consequently have occurred, particularly because the main outcomes, including health‐promoting lifestyle and PWB, were self‐reported. Detection bias cannot be completely excluded for the same reason, as outcome assessment depended on participants’ responses. Where feasible, future studies could use attention‐control conditions or alternative educational interventions with different aims to reduce participants’ awareness of their group allocation. Finally, relatively few studies have examined populations, outcomes, and family‐centered intervention protocols closely comparable to those in the present study. This makes direct replication and comparison across studies difficult. Multicenter studies with standardized intervention protocols and longer follow‐up periods are needed to examine the reproducibility and external validity of these findings.

### 5.2 Implications for Practice and Future Research

The findings may be relevant to MS care in the Middle East, where family members often provide substantial support with long‐term disease management. Including family caregivers in structured education and empowerment programs may allow support for MS self‐management to continue beyond the clinical setting and into patients’ everyday lives. For nursing practice, the findings support the use of family‐centered education as a complementary part of routine MS care, particularly in areas such as lifestyle modification, self‐care, treatment adherence, and psychological adaptation. At the same time, implementation in other settings needs to take account of differences in healthcare systems, family structures, cultural expectations, health literacy, and access to professional resources. Multicenter and cross‐cultural randomized trials are needed to determine whether the findings observed here can be reproduced in other populations and healthcare settings. Future research should also examine longer‐term outcomes, the role of booster interventions, cost‐effectiveness, and the suitability of validated outcome measures in different cultural settings. In this study, a structured FCEM‐based intervention involving patients with MS and their primary family caregivers was associated with greater improvements in health‐promoting lifestyle and PWB than routine care alone. Family‐centered empowerment may therefore be useful as a complementary educational approach in MS management. Larger multicenter studies with longer follow‐up are needed to determine the durability of these changes and their applicability to other populations.

## 6 Conclusion

The findings of this randomized controlled clinical trial indicate that the FCEM can positively influence health‐promoting lifestyle and PWB among patients with MS. Most of the improvements observed immediately after the intervention were still present at the 2‐month follow‐up. This suggests that involving family members in structured education and empowerment may help patients maintain beneficial self‐management behaviors after the formal intervention has ended. These findings have implications for nursing practice and the delivery of MS care. Involving family members more closely in patient education and supportive services may complement existing care, particularly because family caregivers can help patients maintain health‐promoting behaviors and self‐management strategies at home. Structured family‐centered education may therefore be used as a complementary component of routine care for individuals with MS. Future studies should examine the effectiveness, durability, and sustainability of family‐centered empowerment interventions in larger multicenter samples and in different cultural and healthcare settings. Further research is also needed on the international applicability and psychometric validation of the instruments used to assess health‐promoting behaviors and PWB. Longer‐term outcomes and the potential value of booster educational sessions should also be examined. Such research may help determine whether the present findings can be replicated and how broadly the FCEM can be applied in MS care.

## Author Contributions

M.B. and L.R. conceptualized and organized the study. M.B., R.D., and A.Z. were responsible for data collection and execution. M.B. and A.D. designed and/or executed the statistical analyses. All authors have read and approved the final version of the manuscript. The corresponding author Mostafa Bijani, had full access to all of the data in this study and takes complete responsibility for the integrity of the data and the accuracy of the data analysis.

## Funding

The authors have nothing to report.

## Conflicts of Interest

The authors declare no conflicts of interest.

## Transparency Statement

The corresponding author, Mostafa Bijani, affirms that this manuscript is an honest, accurate, and transparent account of the study being reported; that no important aspects of the study have been omitted; and that any discrepancies from the study as planned (and, if relevant, registered) have been explained.

## Supporting information


Supporting File 1

Supporting File 2

## Data Availability

The data that support the findings of this study are available from the corresponding author upon reasonable request.
