# Supplementary material for: Impact of the Family‐Centered Empowerment Model on Health‐Promoting Lifestyle and Psychological Well‐Being in Patients With Multiple Sclerosis: A Randomized Controlled Clinical Trial
Source: Health Sci Rep. 2026 Oct 26;9(11):e73423. doi: 10.1002/hsr2.73423 (PMC13697530; doi:10.1002/hsr2.73423)
Supplement: Supplementary file 1 [file HSR2-9-e73423-s002.docx]

**Education sessions**

**Stage One: Perceived Threat (Knowledge & Awareness)**

Stage 1 of the aforementioned model intends to raise awareness, enhance attitudes regarding the disease, and foster susceptibility (perceived susceptibility) to complications, and finally motivating them to take action. The goal of perceived severity (awareness and knowledge of the individual about the disease) is for the individual to perceive the disease seriousness and feel threatened by it through recognizing the risks or complications of the disease. The goal of perceived susceptibility (the individual's attitude toward their own illness) is for the individual to grasp their own situation and to have a correct definition of health in light of the disease process and the likelihood of recurrence with its complications and exacerbation of the existing conditions. In order to sensitize and raise the information of the patients and their families, group sessions were held about the nature of multiple sclerosis, types of MS, causes and symptoms of the disease, disease diagnosis, available treatments, drug management (how to take it, drug side effects, and the importance of adhering to medications), diet and nutrition (nutritional tips, effective diets in MS), the significance of regular exercise and physical activity, depression in MS, and practical solutions for psychological health during two sessions (each session lasting 45 to 60 minutes) with educational aids (such as whiteboards, educational booklets, PowerPoint presentations, film screenings, Q&A, and lectures).

**Stage Two: Promoting Self-Efficacy (Skill Acquisition)**

This stage pursues the goal to enhance a person’s belief to be able to do a particular task. For this purpose, problem-solving sessions (two sessions lasting 45 to 60 minutes) were held for the patients and their families individually and in groups (groups of two to three individuals). During these above mentioned sessions, the patients and their families were practically exposed to problem demonstrations and the problem-solving process. In these two sessions, the much-needed skills for controlling the disease better were explained and practiced. For instance, one of the nuisances some MS patients challenge with is urinary incontinence, which was demonstrated in the classroom by playing a video of Kegel exercises for the patients and their family members to tighten pelvic muscles and control urinary incontinence. Muscle weakness and imbalance are also common in MS patients, so they were taught active and passive exercises (stretching exercises such as raising legs or arms, balance boosting exercises such as standing on one leg, moving in a straight line) to tighten muscles, boost balance and coordination, reduce fatigue, and improve daily functioning. Then, the patients and families were required to perform and explain this skill.

**Stage Three: Self-belief and Self-esteem Improvement (Educational Participation)**

Stage three of the empowerment model motivates active participation, in which the patient teaches the material discussed in the previous sessions to their family during a session and the researcher provided help and guidance, if necessary. It targets to increase the patients' self-esteem due to observing their capacity to provide information to family members and support the family. Transferring educational contents to the family by the patients brought about better patient learning and observing their potential to execute an educational program improved the patient's self-esteem and self-belief. Besides, the researcher realized 1- How successful the patients were in transferring educational contents. 2- The points the patients did not transfer to the family caregivers were discussed. 3- In case the patients and family caregivers had any questions, their questions were answered. 4- The researcher also asked the patients to demonstrate the patients' self-efficacy relevant skills (including how to do exercises). If the patient and family caregivers were unable to transfer that skill correctly, it was practically elaborated to them again.

**Stage Four: Evaluation**

Evaluation consists of 2 steps including 1: continuous (formative) evaluation and 2: a final(summative)assessment by questionnaires and checklists:

**1- Formative evaluation**: During the empowerment sessions, some oral questions were posed about the content of the first stage sessions and their scores were calculated. The evaluation of self-efficacy sessions was done practically and by correctly performing the trained skills. And the third stage (self-esteem) was evaluated by examining the patient's level of cooperation and interest in educational participation.

**2- Summative evaluation**: Immediately after the empowerment sessions got over, the psychological well-being (PWB) and health-promoting lifestyle profile (HPLP) scales were completed, and two months later, the above mentioned scales got refilled.

Follow-up and telephone calls were made to answer their questions. The questions posed by the patients and family caregivers were mostly about medication, diet, exercise and stress management, for which the research team fully answered their questions and the necessary guidance was provided, and overall 35 telephone-based sessions were held
